# Curability of metastatic cancer: a survey of medical oncologists

**DOI:** 10.1093/jncics/pkaf115

**Published:** 2025-12-05

**Authors:** Shalini Subramaniam, Kim Tam Bui, Martin R Stockler, Belinda E Kiely

**Affiliations:** NHMRC Clinical Trials Centre, University of Sydney, Camperdown, NSW, Australia; Bankstown-Lidcombe Hospital, Bankstown, NSW, Australia; Concord Cancer Centre, Concord, NSW, Australia; NHMRC Clinical Trials Centre, University of Sydney, Camperdown, NSW, Australia; Concord Cancer Centre, Concord, NSW, Australia; NHMRC Clinical Trials Centre, University of Sydney, Camperdown, NSW, Australia; Concord Cancer Centre, Concord, NSW, Australia; Macarthur Cancer Therapy Centre, Campbelltown, NSW, Australia

## Abstract

**Background:**

Patients with metastatic cancer are living longer due to treatment advances. We explored oncologists’ perceptions about curability in metastatic cancer.

**Methods:**

We invited medical oncologists to complete a 21-item online survey. We conducted descriptive analyses and thematically analyzed free-text responses.

**Results:**

A total of 126 respondents completed the survey. The median age was 39 years (range = 27-75). Most respondents worked in Australian (64%), metropolitan (88%), public practices (56%). The most frequently treated cancer types were breast (55%), lung (52%), and colorectal (50%). In total, 82% reported thinking that patients with metastatic cancer can be cured. Cancer types with the highest perceived chance of cure (median percentage) were testicular (81%), melanoma (32%), and colorectal (16%). At the time of diagnosis of metastatic cancer, 51% reported they would tell a patient that cure was possible. After treatment, 29% reported telling some patients they *had been* cured, whereas 74% reported telling some patients that they *may have* been cured. A higher proportion *thought* cure was a realistic possibility with immunotherapy (83%) rather than chemotherapy (40%), but only 44% and 27%, respectively, reported they would *tell* this to patients. In total, 46% reported discussing the possibility of cure more frequently with immunotherapy, 5% more frequently with chemotherapy, 7% as frequently with both, and 42% not discussing with either. Respondents identified oligometastatic disease, actionable mutations, and durable responses to immunotherapy as factors associated with cure.

**Conclusions:**

Most respondents reported thinking that metastatic cancer *is* curable but were reluctant to tell individual patients with metastatic cancer they *had been* cured.

## Introduction

The idea of *cure* is complex, particularly in oncology.^1^ Mechanistically, cure can be conceptualized as the complete eradication of malignancy in an individual with no possibility of recurrence. However, practical definitions are more complicated and vary according to cancer type and extent.

In localized, early-stage cancer, the term *cure* is sometimes used when cancer has not recurred 5 or more years after treatment,^2^ or when a person dies from a cause other than their cancer. Another proposed definition, suitable for groups rather than individuals, is when the mortality rate in a group with a certain cancer returns to the rate expected in the general population of the same sex and age.^3^

These definitions are not readily transferrable to metastatic cancer, where no accepted definition of cure exists.^4^ The term is especially contentious because systemic anticancer treatments were initially cytotoxic, aimed at reducing tumor size and growth, relieving symptoms, and thereby improving quality and length of life. Cure was neither a typical goal nor an expected outcome of cytotoxic therapy for most people with metastatic cancers. As such, oncologists may hesitate to use the term with their patients to avoid giving false hope or fueling unrealistic expectations about cure.^5^

Advances in cancer drug development have substantially shifted the treatment paradigm for metastatic cancer. Immune checkpoint inhibitors, targeted therapies, and antibody-drug conjugates have led to prolonged survival in patients with metastatic cancer.^6-9^ However, not all patients benefit, with considerable variability in outcomes due to tumor biology, patient factors, and access to treatment. This poses an important challenge for oncologists and their patients when considering and discussing the potential benefits and harms of contemporary anticancer treatments, particularly their effects on survival.

Recent literature highlights increasing discussion around long-term survival and the possibility of cure in select metastatic contexts, such as advanced melanoma^10^ and oligometastatic disease.^11^ The term *metavivors* has emerged to describe a new group of people living long-term with metastatic cancer who are not in active decline, akin to those living with complex, chronic illnesses.^12^^,^^13^ Yet even in this evolving conversation, there remains inconsistency in how curability is interpreted and communicated. These findings reveal an unmet need for more comprehensive understanding of how oncologists perceive and discuss cure in different metastatic settings, and highlight the growing complexity of survivorship in this patient population.

In this study, we sought to explore the perceptions and attitudes of medical oncologists regarding curability in metastatic cancer, and how these perspectives influence their communication with patients.

## Methods

### Study design

We conducted a cross-sectional survey of medical oncologists and medical oncology trainees treating solid tumors. The study protocol was approved by the University of Sydney, Human Research Ethics Committee (2024/HE000238).

### Study questionnaire

We developed a 21-item online questionnaire to explore oncologists’ perspectives on the curability of metastatic cancer and the extent to which they discuss the possibility of cure with their patients (Appendix 1). The survey was structured to first explore general beliefs about curability, followed by questions probing specific clinical contexts and preferences for communication and prognostic disclosure.

Survey questions were developed by the study team based on gaps in the literature and areas of interest relevant to prognostic communication in metastatic cancer. To assess face validity, select survey items were discussed virtually with a group of 9 early to mid-career medical oncologists. Feedback was sought on interpretation of key terms such as *cure* and on how the questions were understood and answered. This informed iterative refinements to improve clarity and content validity. The final online instrument was pilot-tested by the study team, which included 4 medical oncologists, to assess readability, logic, and technical functionality before wider dissemination.

We asked respondents whether they believed metastatic cancer is curable and what they would tell patients about the possibility of cure. To contextualize this section of the survey, an introductory text outlined some commonly cited definitions of cure in oncology. Respondents were asked to estimate the percentage of patients with distant metastases they think could be cured for each of 14 specific cancer types using visual analogue scales (0%-100%). Respondents were also presented with a series of simple clinical scenarios and were asked when they think a patient with metastatic cancer has been cured based on time on or after stopping treatment without cancer progression.

We included several questions on immunotherapy, given its increasing use across several metastatic cancer subtypes and emerging evidence of durable responses and long-term survivors. Respondents were asked if they discuss the possibility of cure differently when explaining the benefits of immunotherapy compared with chemotherapy to patients with metastatic cancer, whether they *think* cure is a realistic possibility, and if they would *tell* patients that cure is a possibility with each of these treatments.

They were also asked when they discuss stopping treatment in patients with 4 types of metastatic cancer whose cancer has not progressed while receiving treatment with novel and targeted therapies: kidney cancer and immunotherapy, melanoma and immunotherapy, breast cancer and HER2-targeted therapy, and oncogene-addicted lung cancer and targeted therapy.

To understand how respondents provide information about prognosis (expected survival time) to patients at the start of immunotherapy, we asked them to select from a list of communication approaches including quantitative estimates, time ranges, probabilities, and best-case and worst-case scenarios.

An optional, open-ended question invited respondents to describe tumor or clinical characteristics they considered important when thinking about cure in metastatic cancer. A final free-text item provided respondents the opportunity to share additional views or comments on the topics explored in the survey.

### Recruitment of respondents

We invited medical oncologists, clinical oncologists (dual training in medical oncology and radiation oncology), and medical oncology trainees to complete the survey through Australian professional medical networks including the Medical Oncology Group of Australia, the national professional organization representing Australian medical oncologists, and the Australia & Asia Pacific Clinical Oncology Research Development alumni network. The survey was promoted internationally via social media platforms including X (formerly Twitter), LinkedIn, and Facebook. Professional networks were encouraged to distribute the study invitation via newsletters. Additional recruitment strategies included snowball sampling, whereby initial recipients were encouraged to forward the survey invitation to interested colleagues. A reminder was sent through the mailing lists and through reposting on social media platforms. Completion of the survey was taken to indicate informed consent to participate in the study. Data were collected electronically and stored securely using REDCap (version 14.3.14). All data were anonymous and nonidentifiable. All respondents who completed the full survey were included in this analysis.

A target sample size of 100 participants was prespecified to provide 95% confidence intervals no wider than ±10% for proportions and approximately ±0.20 standard deviations for means. Statistical analyses and data visualizations were conducted using RStudio (version 2024.12.0 + 467). Free-text responses were reviewed by the study team and analyzed thematically to identify recurring concepts, which were then categorized under key themes.

## Results

A total of 126 participants completed the survey between September and October 2024, including 119 consultant medical or clinical oncologists and 8 medical oncology trainees. Only 1 respondent was excluded due to partial survey completion, where only some demographic questions were completed.

Respondent characteristics are summarized in Table 1. The median age was 39 years (range = 27-75), and 51% were women. Most worked in Australia or New Zealand (74%), in metropolitan centers (88%), and practiced predominantly in the public sector (56%). Time since obtaining oncology qualifications was <5 years in 36%, 5-10 years in 24%, and >10 years in 34%; 6% were oncology trainees. The cancer types treated by the highest percentages of respondents were breast (55%), lung (52%), and colorectal (50%) cancers.

**Table 1.** Baseline characteristics (N = 126).

Characteristic	n (%)
Median age, years (range)	39 (27-75)
Gender	
Woman	64 (51)
Man	62 (49)
Country of practice	
Australia	79 (63)
New Zealand	14 (11)
India	11 (9)
Singapore	5 (4)
Malaysia	3 (2)
Other	14 (11)
Practice type	
Public	70 (56)
Private	20 (16)
Public and private	36 (29)
Practice location	
Metropolitan	111 (88)
Rural/regional	15 (12)
Clinical experience	
<5 years	44 (35)
5–10 years	31 (25)
>10 years	43 (34)
Advanced trainee	8 (6)
Cancer types routinely treated	
Breast	69 (55)
Lung	65 (52)
Colorectal	63 (50)
Genitourinary	61 (48)
Upper gastrointestinal	51 (40)
Gynecological	38 (30)
Head and neck	34 (27)
Melanoma	33 (26)
Sarcoma	26 (23)
Other	29 (21)

Data are *n* (%) unless otherwise specified.

### Oncologists’ perceptions about cure in metastatic cancer

Eighty-two percent of respondents reported thinking that patients with metastatic cancer can be cured. The cancer types with the highest reported percentage chance of cure were testicular (81%), melanoma (32%), and colorectal (16%) cancers, and those with the lowest perceived likelihood were stomach (3%), mesothelioma (2%), and esophageal (2%) cancers (Table 2).

**Table 2.** Reported percentage chance of being cured, by type of metastatic cancer (*n* = 126).

Type of metastatic cancer	Median %	IQR %
Testicular	81	71-88
Melanoma	32	20-50
Colorectal	16	9-25
Kidney	13	6-24
Lung	13	6-21
Breast	7	4-14
Ovarian	7	1-12
Endometrial	6	0-12
Prostate	6	0-14
Bladder	5	0-12
Head and neck	5	0-11
Stomach	3	0-7
Mesothelioma	2	0-7
Esophageal	2	0-7

Respondents rated the chance of being cured for each cancer type on a scale from 0% to 100%. Items could be left unanswered if they did not know the answer or did not treat that cancer type.

Fifty-one percent of respondents reported that they would tell a patient with newly diagnosed metastatic cancer that cure was possible. For patients with metastatic cancer who had been treated, 29% reported that they would tell some patients that they *have been* cured, whereas 74% reported they would tell some patients that they *may have been* cured.

### Clinical scenarios about cure in metastatic cancer

When considering scenarios of patients with metastatic cancer, most respondents (95%) thought patients had been cured when the cancer had not progressed 5 years after stopping treatment (Table 3). Fewer respondents considered patients cured if they remained on treatment and the cancer had not progressed at 1, 2, and 5 years after starting treatment.

**Table3.** Proportion of respondents who considered a patient with metastatic cancer to be cured in different clinical scenarios.

Clinical scenarios	*n* (%)
***When a patient remains on treatment and the cancer has not progressed***	
5 years after starting treatment	21 (17)
2 years after starting treatment	5 (4)
1 year after starting treatment	1 (1)
***When a patient has stopped treatment and the cancer has not progressed***	
5 years after stopping treatment	120 (95)
2 years after stopping treatment	17 (13)
1 year after stopping treatment	2 (2)

Respondents were required to select at least 1 of the 6 possible scenarios. Percentages may exceed 100% because multiple responses were allowed.

When asked about stopping treatment in patients with metastatic cancer whose cancer had not progressed, respondents would discuss stopping after 2 years with immunotherapy for kidney cancer (64%) and melanoma (63%). In contrast, fewer respondents would discuss stopping treatment with HER2 directed therapy for breast cancer (12%) or a tyrosine-kinase inhibitor for oncogene-addicted lung cancer (5%). Substantial proportions reported they would never discuss stopping HER2 directed treatment for metastatic breast cancer (44%) or a tyrosine-kinase inhibitor for oncogene-addicted lung cancer (69%) (Table 4).

**Table 4.** Proportion of respondents who would discuss stopping treatment in patients with stable metastatic cancer, by cancer type and treatment.

	Cancer type and treatment (*n*, %)			
Scenario	Kidney cancer – immunotherapy	Melanoma – immunotherapy	Breast cancer – HER2 therapy	Lung cancer – TKI
	(*n* = 80)	(*n* = 76)	(*n* = 84)	(*n* = 80)
>1 year since starting treatment	1 (1)	9 (12)	2 (2)	0 (0)
>2 years since starting treatment	51 (64)	48 (63)	10 (12)	4 (5)
>5 years since starting treatment	21 (26)	19 (25)	35 (42)	21 (26)
I never discuss stopping treatment	7 (9)	0 (0)	37 (44)	55 (69)

Abbreviations: HER2 = human epidermal growth factor receptor 2; TKI = tyrosine kinase inhibitor.

### Explaining treatment benefits and possibility of cure

A greater proportion responded *thinking* that cure is a realistic possibility with immunotherapy (83%) than with chemotherapy (40%), but only 44% and 27%, respectively, responded that they *tell* patients that cure is a realistic possibility when discussing these treatments.

When talking about immunotherapy compared with chemotherapy, 46% responded that they discussed the possibility of cure more frequently with immunotherapy, 5% responded that they discussed the possibility of cure more frequently with chemotherapy, 7% that they discussed cure as frequently with both treatments, and 42% that they did not discuss cure with either.

When asked about prognosis by patients starting immunotherapy for metastatic cancer, most respondents preferred using multiple time ranges with accompanying probabilities (68%), followed by a probability of surviving a given length of time (39%) and a range of lengths of time (26%) (Table 5).

**Table 5.** Preferred ways to answer questions about prognosis from patients with metastatic cancer starting immunotherapy.

Methods	No. (%)
Multiple ranges of time with probabilities, for example, best-case, typical-case, worst-case scenarios for survival time	86 (68)
A probability of surviving a given length of time, for example, 20% 5-year survival	49 (39)
A range of lengths of time, for example, “6 to 12 months”	33 (26)
A specified length of time, for example, median survival in months	23 (18)
A range of units of time without numbers, for example, “weeks to months”	20 (16)
A unit of time without numbers, for example, “months,” “years”	15 (12)
The probability of being cured	12 (10)
No quantitative estimate	1 (1)
I have not been in this situation	1 (1)
Other	5 (4)

Multiple responses allowed.

### Characteristics deemed important when thinking about cure

Factors that respondents considered important when thinking about curability in metastatic cancer were categorized into 4 key themes: cancer subtype, biomarkers, treatment approaches, and patient-related factors (Table 6).

**Table 6.** Tumor and clinical characteristics that respondents deemed important when thinking about curability.

Key theme	Factors associated with increased likelihood of cure
Cancer subtype and disease burden	Cancers considered highly curable (eg, testicular and other germ cell tumors, melanoma) Oligometastatic disease or limited metastatic burden amenable to surgery or ablative therapy (eg, resection or ablation of metastases and primary disease in oligometastatic colorectal cancer) Location of metastasis (eg, nodal vs visceral)
Molecular and imaging biomarkers	Undetectable circulating tumor DNA Actionable driver mutations associated with durable responses (eg, ALK, EGFR, HER2) Markers of response to immunotherapy (eg, PD-L1 expression, high TMB, MSI-high status) Early complete metabolic response on PET imaging
Treatment-related factors	Durable responses observed with immunotherapy Curative potential of chemotherapy in select cancers (eg, germ cell tumors) Benefit from multimodal approaches integrating systemic and local therapies
Patient-related factors	Good performance status and overall fitness for therapy Absence of significant comorbidities

Abbreviations: ALK = anaplastic lymphoma kinase. EGFR = epidermal growth factor receptor. HER2 = human epidermal growth factor receptor 2. PD-L1 = programmed death-ligand 1. TMB = tumor mutational burden. PET = positron emission tomography.

Respondents also reflected on the conceptual challenges of defining cure and how they approach this in patient conversations. Many expressed reluctance to use the term *cure*, instead favoring wording such as “remission” or “no evidence of disease,” due to the risk of late relapse and uncertainties around long-term disease control. The concept of cure was typically avoided at diagnosis and introduced only after a sustained period of response.

## Discussion

In this survey of medical oncologists and trainees, we found that most respondents believed that there are patients whose metastatic cancer can be cured (82%) and that cure was more likely with immunotherapy (83%) than with chemotherapy (40%). However, most respondents seemed hesitant to tell patients that their cancer had been cured (27%-44%). Nearly all respondents (95%) would consider a patient with metastatic cancer cured when the cancer has not progressed 5 years after stopping cancer treatment. When providing information about prognosis to patients starting immunotherapy, most respondents reported a preference for using multiple scenarios for survival (68%) rather than single point estimates.

These findings reflect the impact of an evolving therapeutic landscape on oncologists’ perceptions about curability in metastatic cancer. There was an expected discrepancy between respondents’ belief in curability and their actual communication of this with patients. Although optimism exists regarding the curative potential of immunotherapy and other novel agents, this appears tempered in clinical conversations. To our knowledge, few prior studies have directly examined how oncologists perceive curability in metastatic cancer in the era of immunotherapy, highlighting the novelty and relevance of these findings.

Historically, only a few metastatic cancers, such as testicular and other germ cell tumors, have been considered curable with chemotherapy.^14^ For most other cancers, metastatic disease has been associated with inevitable treatment resistance, progression, and death. However, the advent of newer therapies such as immunotherapy has challenged this narrative, with durable responses and long-term survival. This has prompted a rethinking of the binary notion of curable vs incurable, instead highlighting a spectrum of survivorship. Such outcomes are, however, poorly predicted at the start of treatment, and determining the trajectory and long-term outcomes for an individual remains a challenge.

Kaplan-Meier survival curves from pivotal immunotherapy trials often exhibit a distinct horizontal plateau in the tail, frequently interpreted as evidence of cure in a subset of patients.^15^ However, such plateaus may reflect incomplete follow-up, and they are also seen in treatments regarded as noncurative such as chemotherapy.

Communicating the breadth and the uncertainty of these outcomes adds complexity to clinical discussions. These ambiguities likely contribute to restraint when oncologists discuss treatment intent and prognosis, with cure often being considered a retrospective description rather than a promise about the future. The preference for presenting multiple scenarios for survival may reflect efforts to balance hope with honesty and to avoid fueling unrealistic expectations.^1^^,^^16^

The time point at which respondents discussed stopping treatment in the absence of disease progression varied across the 4 tumor types and treatment modalities evaluated. For immunotherapy-treated cancers, the 2-year mark emerged as a common threshold, likely reflecting durations used in landmark trials shaping clinical practice and local reimbursement guidelines. Many oncologists feel comfortable discontinuing immunotherapy after this time given the lack of evidence supporting treatment continuation beyond protocol-defined periods. In contrast, chemotherapy and targeted therapies have typically been administered until disease progression or unacceptable toxicity based on the principle that these treatments control rather than eradicate cancer. Of note, the STOP-HER2 Trial is being conducted to evaluate if anti-HER2 treatment can be safely stopped in patients with HER2-positive metastatic breast cancer who have had exceptional response to treatment, defined here as individuals free of disease progression after at least 3 years of first-line treatment.^17^

## Strengths and limitations

Our study has several strengths. We asked oncologists directly about their thoughts and personal beliefs about curability in metastatic cancer and presented questions in varied ways to probe this idea. We included a range of tumor types and treatments to capture general perspectives, while also focusing on novel therapies that are redefining long-term survival in patients with metastatic cancer. The structured survey design facilitated broad and efficient collection of perspectives from a geographically diverse cohort of oncologists. The majority of respondents were from Australia and New Zealand; participants from other countries increased the range of perspectives and experiences. Our findings provoke thinking about a concept that is important but remains controversial and under-researched.

There are limitations that should also be considered. All surveys are vulnerable to selection bias; those who responded may have different views from those who did not and may have been motivated by strong views or familiarity with the topic. Respondents skewed younger, with a median age of 39 years, reflecting a cohort of early- and mid-career oncologists, and their level of clinical experience may have influenced responses. The predominance of respondents from higher-income countries may limit generalizability, particularly given global disparities in access to novel therapies that influence perceptions of curability. Our inclusion of testicular cancer among the 14 tumor types specifically asked about may have inadvertently influenced responses to later questions. Most questions required selection of predefined responses, which did not allow for nuance. Our approach was exploratory and hypothesis-raising, and the purpose of this survey was to identify gaps and directions for further research.

## Implications for clinical practice and future research

Our study highlights the need for clearer and consistent language when discussing treatment intent and long-term outcomes in metastatic cancer. The term *cure* remains difficult to define, particularly in its application to individuals, and the apparent hesitancy of our respondents to use *cure* in conversations with patients reflects this uncertainty. From the patient’s perspective, ambiguous or inconsistent terminology may lead to confusion and unrealistic expectations^18^^,^^19^ and may influence treatment decisions in ways that do not align with their personal values or goals.^20^

Although definitions of *cure* and *survivorship* vary across the literature, the lack of consensus and absence of standardized language are again highlighted by our findings. This is especially important given that patients with metastatic cancer are increasingly living longer and will be asking the same questions this survey set out to explore. Patients may accept they are not cured, but although their cancer is well controlled and they are not actively dying, they must contend with prolonged uncertainty.^21^ Unsurprisingly, information and psychological domains, including understanding prognosis and coping with uncertainty, consistently rank among the most significant needs for patients with advanced cancer and their caregivers.^22^

Future research should seek to define curability more clearly and develop practical frameworks to help oncologists navigate these complex conversations. Although this survey explored oncologists’ perspectives, further research is also needed to understand what patients think *cure* means and how they would like oncologists to discuss the possibility of *cure*. It is important to understand what language best suits the informational and emotional needs of patients. Focus groups, interviews, and surveys with patients may help identify important nuances and novel methods for framing these conversations. Establishing a shared understanding is essential for improving discussions about prognosis and ensuring patient-centered care that sustains hope without compromising truthfulness.

## Conclusion

Most oncologists responding to our survey believed cure is possible in metastatic cancer, particularly with immunotherapy-responsive cancers, and yet many were hesitant to tell patients that their cancer had been cured even if they considered it possible. Uncertainties about the definition of cure and an individual’s likelihood of cure may contribute to this cautious communication, but such ambiguity may also confuse patients. Clearer and more accurate terminology is needed to improve discussions about prognosis and long-term outcomes in metastatic cancer.

## Supplementary Material

pkaf115_Supplementary_Data

## Data Availability

The data underlying this article cannot be shared due to the privacy of individuals who participated in the study. Individual deidentified data can be made available from the repository to accredited researchers who submit a proposal that is approved by the NHMRC Clinical Trials Centre.
