# Supplementary material for: Curability of metastatic cancer: a survey of medical oncologists
Source: JNCI Cancer Spectr. 2025 Dec 5;10(1):pkaf115. doi: 10.1093/jncics/pkaf115 (PMC12783893; doi:10.1093/jncics/pkaf115)
Supplement: pkaf115_Supplementary_Data [file pkaf115_supplementary_data.pdf]

Is Metastatic Cancer Curable?

We invite you to participate in this survey: Is Metastatic Cancer Curable?

1. What is this study about?

The concept of cure in oncology is complex with no single accepted definition. In early-stage cancer, cure is typically defined as the complete eradication of cancer from a person's body with no likelihood of its return. In metastatic cancer, the use of the term "cure" is far more challenging. Most metastatic cancers are considered incurable with chemotherapy. While palliative chemotherapy is used to control tumour growth and alleviate cancer-related symptoms, the chance of complete eradication of cancer with chemotherapy is minimal.

Advances in oncology drug development has led to more effective anticancer treatments, such as immune checkpoint inhibitors and targeted therapies, which have substantially improved overall survival for patients with metastatic cancer.

This has led to unique challenges for both oncologists and patients with metastatic cancer when discussing the benefits of anticancer treatments, their effects on overall survival and the potential for cure.

We aim to determine oncologists' perceptions about survival and the possibility of cure in metastatic cancer.

2. Who is running the study?

We are researchers from the NHMRC Clinical Trials Centre, The University of Sydney. The study team members are:

Dr Belinda Kiely Chief Investigator

Dr Shalini Subramaniam Co-investigator, PhD Candidate

Dr Kim Tam Bui Co-investigator

Prof Martin Stockler Co-investigator

Dr Shalini Subramaniam is conducting this study as part of her PhD undertaken at The University of Sydney.

3. Who can take part in the study?

Medical/clinical oncologists and oncology trainees.

4. What does participation in this research involve?

This survey will take no longer than 15 minutes to complete and can be accessed via the link below on a desktop or mobile device.

5. Can I withdraw once I've started?

Participation is voluntary. By submitting your survey, you are consenting to partake part in the study. You have the option to withdraw any time before submitting. Your decision will have no impact on your current or future relationship with the researchers or anyone else at The University of Sydney.

6. Are there any risks or costs?

Aside from giving up your time, we do not expect that there will be any risks or costs associated with participating in this study.

7. Are there any benefits?

There are no direct benefits for you as a participant in this study.

8. What will happen to information that is collected?

All data collected will be anonymous and deidentified. We plan to publish the findings of this study. You will not be identifiable in these publications.

9. Will I be told the results of the study?

A summary of the results will be made available via MOGA's newsletter and on their website.

10. What if I would like further information?

If you have further questions about this study, please contact

Dr Shalini Subramaniam on behalf of Dr Belinda Kiely (Chief Investigator)

Medical Oncologist and PhD Candidate

NHMRC Clinical Trials Centre, University of Sydney

Email: shalini.subramaniam@sydney.edu.au

11. What if I have a complaint or any concerns?

The ethical aspects of this study have been approved by the Human Research Ethics Committee (HREC) of The University of Sydney (2024/HE000238) in accordance with the National Statement on Ethical Conduct in Human Research (2007).

If you have any concerns about the study's procedures or would like to make a complaint to someone not involved in the study, please contact the University:

Human Ethics Manager

human.ethics@sydney.edu.au

+61 2 8627 8176

Consent

Please select one option.

☐ I confirm that I have read and understood the information provided above.
☐ I do not wish to continue.

Demographics

1 Age __________________________________

2 Gender
◯ Woman
◯ Man
◯ I identify my gender as
◯ Prefer not to say

____

3 Country of practice

- ◯ Australia
- ◯ Afghanistan
- ◯ Albania
- ◯ Algeria
- ◯ Andorra
- ◯ Angola
- ◯ Antigua & Deps
- ◯ Argentina
- ◯ Armenia
- ◯ Austria
- ◯ Azerbaijan
- ◯ Bahamas
- ◯ Bahrain
- ◯ Bangladesh
- ◯ Barbados
- ◯ Belarus
- ◯ Belgium
- ◯ Belize
- ◯ Benin
- ◯ Bhutan
- ◯ Bolivia
- ◯ Bosnia Herzegovina
- ◯ Botswana
- ◯ Brazil
- ◯ Brunei
- ◯ Bulgaria
- ◯ Burkina
- ◯ Burundi
- ◯ Cambodia
- ◯ Cameroon
- ◯ Canada
- ◯ Cape Verde
- ◯ Central African Rep
- ◯ Chad
- ◯ Chile
- ◯ China
- ◯ Colombia
- ◯ Comoros
- ◯ Congo
- ◯ Congo {Democratic Rep}
- ◯ Costa Rica
- ◯ Croatia
- ◯ Cuba
- ◯ Cyprus
- ◯ Czech Republic
- ◯ Denmark
- ◯ Djibouti
- ◯ Dominica
- ◯ Dominican Republic
- ◯ East Timor
- ◯ Ecuador
- ◯ Egypt
- ◯ El Salvador
- ◯ Equatorial Guinea
- ◯ Eritrea
- ◯ Estonia
- ◯ Ethiopia
- ◯ Fiji
- ◯ Finland
- ◯ France
- ◯ Gabon
- ◯ Gambia
- ◯ Georgia
- ◯ Germany
- ◯ Ghana
- ◯ Greece
- ◯ Grenada
- ◯ Guatemala
- ◯ Guinea

◯ Guinea-Bissau
◯ Guyana
◯ Haiti
◯ Honduras
◯ Hungary
◯ Iceland
◯ India
◯ Indonesia
◯ Iran
◯ Iraq
◯ Ireland {Republic}
◯ Israel
◯ Italy
◯ Ivory Coast
◯ Jamaica
◯ Japan
◯ Jordan
◯ Kazakhstan
◯ Kenya
◯ Kiribati
◯ Korea North
◯ Korea South
◯ Kosovo
◯ Kuwait
◯ Kyrgyzstan
◯ Laos
◯ Latvia
◯ Lebanon
◯ Lesotho
◯ Liberia
◯ Libya
◯ Liechtenstein
◯ Lithuania
◯ Luxembourg
◯ Macedonia
◯ Madagascar
◯ Malawi
◯ Malaysia
◯ Maldives
◯ Mali
◯ Malta
◯ Marshall Islands
◯ Mauritania
◯ Mauritius
◯ Mexico
◯ Micronesia
◯ Moldova
◯ Monaco
◯ Mongolia
◯ Montenegro
◯ Morocco
◯ Mozambique
◯ {Burma}
◯ Namibia
◯ Nauru
◯ Nepal
◯ Netherlands
◯ New Zealand
◯ Nicaragua
◯ Niger
◯ Nigeria
◯ Norway
◯ Oman
◯ Pakistan
◯ Palau
◯ Panama
◯ Papua New Guinea
◯ Paraguay
◯ Peru
◯ Philippines
◯ Poland

◯ Portugal
◯ Qatar
◯ Romania
◯ Russian Federation
◯ Rwanda
◯ St Kitts & Nevis
◯ St Lucia
◯ Saint Vincent & the Grenadines
◯ Samoa
◯ San Marino
◯ Sao Tome & Principe
◯ Saudi Arabia
◯ Senegal
◯ Serbia
◯ Seychelles
◯ Sierra Leone
◯ Singapore
◯ Slovakia
◯ Slovenia
◯ Solomon Islands
◯ Somalia
◯ South Africa
◯ South Sudan
◯ Spain
◯ Sri Lanka
◯ Sudan
◯ Suriname
◯ Swaziland
◯ Sweden
◯ Switzerland
◯ Syria
◯ Taiwan
◯ Tajikistan
◯ Tanzania
◯ Thailand
◯ Togo
◯ Tonga
◯ Trinidad & Tobago
◯ Tunisia
◯ Turkey
◯ Turkmenistan
◯ Tuvalu
◯ Uganda
◯ Ukraine
◯ United Arab Emirates
◯ United Kingdom
◯ United States
◯ Uruguay
◯ Uzbekistan
◯ Vanuatu
◯ Vatican City
◯ Venezuela
◯ Vietnam
◯ Yemen
◯ Zambia
◯ Zimbabwe

---

4 Please select the current Australian State or Territory in which you are practicing.

◯ New South Wales
◯ Queensland
◯ Victoria
◯ Tasmania
◯ South Australia
◯ Western Australia
◯ Australian Capital Territory
◯ Northern Territory

5 My oncology practice predominantly in the:

◯ Public sector
◯ Private sector
◯ Public and private sector

6 Which of the following applies to your primary place of work?

◯ Metropolitan centre
◯ Rural/ regional centre

7 Please select the option below that best reflects your experience treating cancer.

◯ < 5 years since obtaining FRACP or equivalent
◯ 5 - 10 years since obtaining FRACP
◯ > 10 years since obtaining FRACP
◯ Advanced trainee

8 Which of the following cancers do you routinely treat (check all applicable)?

☐ Breast
☐ Colorectal
☐ Genitourinary
☐ Gynaecological
☐ Head and Neck
☐ Lung
☐ Melanoma
☐ Sarcoma
☐ Upper Gastrointestinal
☐ Other

Please specify the cancer(s) that you routinely treat

____

9 In the last 3 months, approximately how many patients with metastatic cancer did you start on immunotherapy?

____
(Please provide an estimate)

Oncologists' opinion about cure in metastatic cancer

The concept of cure in oncology is complex and there is no single accepted definition. In early stage cancer, the term is sometimes used when a person's cancer has not returned for at least 5 years after treatment. Other definitions of cancer cure include: when the mortality rate of people diagnosed with a certain cancer returns to the rate expected in the general population of the same sex and age and; when a person dies from a cause other than their cancer.

The following questions will help us understand how you think about cure in patients with metastatic cancer.

10 Do you think patients with metastatic cancer can be cured?

◯ Yes
◯ No

11 For each of the following cancer types, what percentage of patients with distant metastases do you think can be cured?

Select 0 if you think cure is not possible. Leave unanswered if you do not know. Hit reset to clear an answer you have unintentionally entered.

Breast

0% 50% 100%

(Place a mark on the scale above)

Bladder

0% 50% 100%

(Place a mark on the scale above)

Colorectal	0% 50% 100% (Place a mark on the scale above)
Endometrial	0% 50% 100% (Place a mark on the scale above)
Head and neck	0% 50% 100% (Place a mark on the scale above)
Kidney	0% 50% 100% (Place a mark on the scale above)
Lung	0% 50% 100% (Place a mark on the scale above)
Melanoma	0% 50% 100% (Place a mark on the scale above)
Mesothelioma	0% 50% 100% (Place a mark on the scale above)
Oesophageal	0% 50% 100% (Place a mark on the scale above)
Ovarian	0% 50% 100% (Place a mark on the scale above)
Prostate	0% 50% 100% (Place a mark on the scale above)
Stomach	0% 50% 100% (Place a mark on the scale above)
Testicular	0% 50% 100% (Place a mark on the scale above)

12 Please comment on any tumour or clinical characteristics that are important when thinking about cure in patients with metastatic cancer.

____

13 Do you ever tell patients who have been newly diagnosed with metastatic cancer that cure is possible?

◯ Yes
◯ No

14 Do you tell some patients who have been diagnosed with metastatic cancer and received treatment that they have been cured?

- ◯ Yes
- ◯ No

15 Do you tell some patients who have been diagnosed with metastatic cancer and received treatment that they may have been cured?

- ◯ Yes
- ◯ No

16 Consider the following scenarios for patients with metastatic cancer. In which scenarios do you think the patient has been cured?

Select all that apply.

- ☐ when the cancer has not progressed 5 years after stopping cancer treatment
- ☐ when the cancer has not progressed 2 years after stopping cancer treatment
- ☐ when the cancer has not progressed 1 year after stopping cancer treatment
- ☐ when the patient remains on treatment and the cancer has not progressed 5 years after starting treatment
- ☐ when the patient remains on treatment and the cancer has not progressed 2 years after starting treatment
- ☐ when the patient remains on treatment and the cancer has not progressed 1 year after starting treatment

17 For patients with metastatic cancer whose cancer has not progressed while receiving the following treatments, when would you discuss stopping treatment?

	≥5y since starting treatment	≥2y since starting treatment	≥1y since starting treatment	I never discuss stopping treatment	I do not treat this cancer
Breast cancer - HER2 targeted treatment	◯	◯	◯	◯	◯
Oncogene addicted lung cancer - targeted therapies (e.g. tyrosine kinase inhibitors)	◯	◯	◯	◯	◯
Kidney cancer - immune checkpoint inhibitors	◯	◯	◯	◯	◯
Melanoma - immune checkpoint inhibitors	◯	◯	◯	◯	◯

Explaining treatment benefits and the possibility of cure with patients

18 Do you think cure is a realistic possibility when discussing the benefits of the following treatments in patients with metastatic cancer?

i. Chemotherapy (with or without non-immunotherapy treatments)

- ◯ Yes
- ◯ No

ii. Immunotherapy (with or without other treatments)

- ◯ Yes
- ◯ No

19 Do you tell patients with metastatic cancer that cure is a realistic possibility when discussing the benefits of the following treatments?

	i. Chemotherapy (with or without non-immunotherapy treatments)	◯ Yes<br>◯ No
	ii. Immunotherapy (with or without other treatments)	◯ Yes<br>◯ No
20	Do you discuss the possibility of cure differently when talking about immunotherapy as compared to chemotherapy to patients with metastatic cancer?	◯ Discuss cure more frequently with immunotherapy<br>◯ Discuss cure more frequently with chemotherapy<br>◯ Discuss cure as frequently with both treatments<br>◯ Do not discuss cure with either treatment
21	When patients with metastatic cancer start immunotherapy and ask for information about prognosis (expected survival time), which of the following ways do you prefer to answer this question?<br><br>Select all options that apply.	☐ No quantitative estimate<br>☐ A unit of time without numbers, e.g. 'months' 'years'<br>☐ A range of units of time without numbers, e.g. 'weeks to months'<br>☐ A probability of surviving a given length of time, e.g. 20% 5 year survival<br>☐ The probability of being cured<br>☐ A specified length of time, e.g. median survival in months<br>☐ A range of lengths of time, e.g. '6 to 12 months'<br>☐ Multiple ranges of time with probabilities, e.g. best-case, typical-case, worst-case scenarios for survival time<br>☐ Other - please specify below<br>☐ I have not been in this situation
	Please describe further if you selected "Other"	__________________________________
	Please provide any additional views, comments or suggestions about the topics covered in this survey.	
